# Risk Profile of Patients with Brushite Stone Disease and the Impact of Diet

**DOI:** 10.3390/nu15184092

**Published:** 2023-09-21

**Authors:** Roswitha Siener, Maria Sofie Pitzer, Jan Speller, Albrecht Hesse

**Affiliations:** 1 University Stone Center, Department of Urology, University Hospital Bonn, 53127 Bonn, Germany; 2 Department of Medical Biometry, Informatics and Epidemiology, Medical Faculty, University of Bonn, 53127 Bonn, Germany

**Keywords:** kidney stones, urolithiasis, nephrolithiasis, brushite, absorptive hypercalciuria, renal tubular acidosis, urinary pH, calcium, oxalate, diet

## Abstract

This study examined the profile of patients and the impact of diet on the risk of brushite stone formation under controlled, standardized conditions. Sixty-five patients with brushite nephrolithiasis were enrolled in the study. Metabolic, dietary, and 24 h urinary parameters were collected under the habitual, self-selected diet of the patients and the balanced mixed, standardized diet. The [^13^C_2_]oxalate absorption, ammonium chloride, and calcium loading tests were conducted. All patients had at least one abnormality on the usual diet, with hypercalciuria (84.6%), increased urine pH (61.5%), and hyperphosphaturia (43.1%) being the most common. Absorptive hypercalciuria was present in 32.1% and hyperabsorption of oxalate in 41.2%, while distal renal tubular acidosis (dRTA) was noted in 50% of brushite stone formers. The relative supersaturation of brushite did not differ between patients with and without dRTA. Among all recent brushite-containing calculi, 61.5% were mixed with calcium oxalate and/or carbonate apatite. The relative supersaturation of brushite, apatite, and calcium oxalate decreased significantly under the balanced diet, mainly due to the significant decline in urinary calcium, phosphate, and oxalate excretion. Dietary intervention was shown to be effective and should be an integral part of the treatment of brushite stone disease. Further research on the role of dRTA in brushite stone formation is needed.

## 1. Introduction

Brushite and carbonate apatite are the most common calcium phosphate stones [1,2]. Although brushite-containing calculi account for only 1.3% of all urinary stones [2], brushite nephrolithiasis is characterized by a recurrence rate of up to 75%, which is higher than most other types of stones [3]. The brushite content of stones has been demonstrated to correlate with their fragility [4]. The particular density and hardness of brushite calculi, resulting in poor response to shock wave lithotripsy, the large stone burden, and the high frequency of stone episodes require repeated surgical interventions to achieve a high stone-free rate [4,5,6,7,8]. The number of surgical procedures is associated with missed workdays and is known to negatively affect health-related quality of life in stone patients [9].

Brushite stone disease is an aggressive form of nephrolithiasis and requires not only a targeted medical and surgical treatment approach but also comprehensive measures for diagnosis and recurrence prevention. The factors and mechanisms driving the formation of brushite stones are not yet fully understood. Hypercalciuria has been reported to be the most common abnormality in 24 h urine of brushite stone formers [7,10]. Additionally, a urine pH in the range of 6.5 to 6.8 may favor brushite crystallization [1]. While the importance of urinary phosphate excretion is still unclear [11], low urine volume and hypocitraturia are urinary factors that may enhance the risk of brushite stone formation [7,11]. However, knowledge on the role of diet in brushite stone disease is limited, and there is a lack of evidence on the urinary risk profile of brushite stone patients under controlled, standardized conditions. The aim of this study was to further characterize anatomical, metabolic, and nutritional risk factors of brushite nephrolithiasis under free and controlled, standardized conditions and to determine the effects of dietary intervention on urinary risk factors for brushite stone formation.

## 2. Materials and Methods

### 2.1. Patients

A total of 65 patients, 19 women and 46 men, with a history of brushite stone formation were enrolled in this study. Patients were referred to the University Stone Center of the Department of Urology at the University Hospital Bonn for inpatient metabolic evaluation under controlled, standardized conditions. Patients with a documented calculus from a recent stone event that contained any amount of brushite at stone analysis were considered for the study. Urinary stone composition was analyzed using Fourier transform infrared spectroscopy (PerkinElmer, Waltham, MA, USA). The exclusion criterion was primary hyperparathyroidism, which is considered a possible cause of hypercalciuria and calcium phosphate stone formation [12,13]. For four weeks before and during the study, patients discontinued dietary supplements and medications that could affect acid–base status or calcium, oxalate, phosphate, and purine metabolism, such as alkali citrate, sodium bicarbonate, L-methionine, thiazides, or allopurinol. Patients did not receive any dietary recommendations and were asked to maintain their habitual dietary patterns before participating in the study. The study was approved by the Ethics Committee of the Medical Faculty of the University of Bonn (430/19). Written informed consent was obtained from each patient.

### 2.2. Study Design

Medical history, anthropometric, metabolic, and 24 h urinary parameters were collected from the stone formers at baseline under their habitual, free-choice diet. The patients kept a 7-day dietary record during their usual diet. Patients described in detail the type and weighed amount of all food consumed. The computer program PRODI 5.3 (Nutri-Science GmbH, Freiburg, Germany) was used to calculate the dietary composition. The oxalate content of all beverages and foods analyzed in our laboratory was included in the database [14,15,16]. Dietary sodium intake was determined by 24 h urinary sodium excretion [17].

In the subsequent phase, patients received a balanced, standardized diet for a period of 11 days [17,18]. The daily water intake through beverages amounted to 2.5 L. The standardized diet, i.e., the daily intake of the specified fluids and food, results in a metabolic steady state after a few days of adaptation, so that constant urine values are achieved [17,18]. Participants collected 24 h urine during their usual, free-choice diet and after 7 days on the balanced mixed, standardized diet. The calcium loading test was conducted on days 2 and 3, the ammonium chloride loading test on day 4, and the [^13^C_2_]oxalate absorption test on days 9 and 10 under controlled, standardized conditions [19].

### 2.3. [^13^C_2_]Oxalate Absorption Test

The gastrointestinal oxalate absorption of the brushite stone formers was determined with the [^13^C_2_]oxalate absorption test [20]. The test was conducted under a standardized diet on two consecutive days. On the morning of the second day, patients received a capsule containing 50 mg sodium [^13^C_2_]oxalate (33.8 mg [^13^C_2_]oxalic acid) in a fasting state. On both days, the patients collected 24 h urine in fractions. The determination of labeled and unlabeled oxalate was performed by gas chromatography–mass spectrometry. Absorption was expressed as a percentage of the labeled oxalate dose. Hyperabsorption of oxalate is defined as intestinal absorption greater than 10% [18,21].

### 2.4. Ammonium Chloride Loading Test

The ammonium chloride loading test was performed to diagnose distal renal tubular acidosis (dRTA) [13,18]. The amount of NH_4_Cl administered was 0.1 g per kg body weight. A cut-off urine pH of 5.4 in the day profile was used to detect incomplete dRTA.

### 2.5. Calcium Loading Test

The oral calcium loading test was performed for diagnosis of the different forms of hypercalciuria [18]. Patients with urinary calcium excretion ≥5 mmol/24 h on the usual diet were included. Patients started fasting the night before shortly after dinner (6 pm), except for 300 mL of distilled water at 8 pm and at 11 pm. On the test day, patients consumed 600 mL of distilled water at 7 am. The first urine sample was collected between 7 and 9 am on the test day (2 h fasting). Subsequently, 1000 mg calcium in a total volume of 300 mL was administered together with a standardized breakfast at 9 am. At 11 am, 300 mL of water was consumed. The second urine sample was collected between 9 am and 1 pm. In the first and second urine samples, creatinine and calcium were determined. Urinary calcium was expressed as a ratio to urinary creatinine. Absorptive hypercalciuria is defined as urine calcium (mmol/L) to creatinine (mmol/L) ≤0.337 in the first and ≥0.564 in the second urine and renal hypercalciuria as urine calcium (mmol/L) to creatinine (mmol/L) ≥0.338 in the first and ≥0.564 in the second urine.

### 2.6. Urinary Parameters

Urinary volume, pH (potentiometry), density (hydrometer), sodium, potassium, and calcium (ion selective electrode), chloride (coulomb metric titration), magnesium (xylidyl-blue reaction), inorganic phosphate (phosphate molybdate reaction), inorganic sulfate (nephelometry), ammonium (ion selective electrode), creatinine (Jaffé reaction), uric acid (enzymatically, uricase), citrate (enzymatically, citrate lyase), and oxalate (enzymatically, oxalate oxidase) concentrations were measured. The ion-activity product of brushite, struvite, calcium oxalate, and uric acid was determined [18,22,23]. Relative supersaturation with respect to brushite, apatite, struvite, calcium oxalate, and uric acid was calculated using the iterative computer program EQUIL2 [24]. The glomerular filtration rate (eGFR) was estimated using the CKD-EPI study equation for adults [25]. Laboratory quality certification was available for each urine parameter.

### 2.7. Statistical Analysis

The non-parametric Mann–Whitney U test for unpaired data was used to compare continuous variables between two groups. Differences within groups before and after intervention were determined with the non-parametric Wilcoxon signed rank test. Categorical variables were compared using Fisher’s exact test and McNemar’s test. Pearson’s chi-squared test was used to assess the distribution of gender according to the different types of hypercalciuria. One-way analysis of variance (ANOVA) was performed for multi-group comparisons of means after checking the requirements with the Kolmogorov–Smirnov and Levene test. Correlations between variables were calculated using Spearman’s rank correlation. All statistical tests were two-sided for the exclusively explorative analysis with a significance level α = 0.05, without taking into account the effects of multiple testing. Statistical analyses were performed using IBM SPSS version 28.0 and 29.0 (SPSS Inc., Chicago, IL, USA).

## 3. Results

### 3.1. Patients

The characteristics of the 65 brushite stone formers, 19 (29%) women and 46 (71%) men, at baseline are depicted in Table 1. The mean age of patients was 41.9 ± 13.0 years (range: 20 to 76 years), and the mean body mass index (BMI) was 26.3 ± 4.3 kg/m^2^. The mean brushite content of the most recent stone was 82.4% ± 20.1% and ranged from 20% to 100%. Pure brushite stones were found in 25 (38.5%) patients, while 40 (61.5%) patients had mixtures of brushite with other constituents. Among all brushite-containing stones, 23 (35.4%) were mixed with calcium oxalate, ranging from 5% to 60%, and 9 (13.8%) were mixed with carbonate apatite, ranging from 10% to 80%. In the 8 (12.3%) brushite-containing stones mixed with calcium oxalate and carbonate apatite, brushite was present between 40% and 70%, calcium oxalate between 10% and 40%, and carbonate apatite between 10% and 30%.

The mean age of patients at the time of the first stone event was 29.9 ± 11.9 years and ranged from 4 to 66 years. In 67.7% of the patients, the stones formed bilaterally, in 32.3% unilaterally. All but one of the patients had undergone surgical intervention for nephrolithiasis. Ureteroscopy was performed at least once in 85.9% of patients, and 82.8% of patients had undergone at least one extracorporeal shock wave lithotripsy (ESWL). In 75.0% of the patients, urinary calculi had passed spontaneously. A positive family history of stone disease was recorded in 50% of patients, with a first-degree relative affected in 70%. Of 64 patients, 12 patients (18.8%) had anatomical abnormalities with kidney cysts (4 patients) and stenosis (4 patients; 2 ureteropelvic junction obstruction/subpelvic stenosis, 1 calyceal infundibular stenosis, and 1 distal urethral stenosis) being the most common, followed by medullary sponge kidney (3 patients). Distal renal tubular acidosis (dRTA) was noted in 50% of the patients. Two patients had the complete form, while the remaining patients had incomplete dRTA.

### 3.2. Urine Composition

The urine composition of the brushite stone formers under their habitual, self-selected diet and the balanced, standardized diet is presented in Table 2. Under the balanced mixed diet, the ion-activity product of brushite and calcium oxalate declined by 26% each. Moreover, the supersaturation of brushite, apatite, and calcium oxalate under the balanced, standardized diet was significantly lower than under the habitual, free-choice diet of the brushite stone formers. The relative supersaturation of apatite declined by 59% under the balanced mixed diet. Urinary excretion of sodium, potassium, calcium, chloride, phosphate, sulfate, uric acid, oxalate, and urine density declined significantly under the balanced, standardized diet. No significant change was observed in any other urine parameters.

Hypercalciuria was the most common abnormality in 24 h urine under the usual diet, affecting 85% of all brushite stone patients (Table 3). Under the habitual, free-choice diet, urinary calcium excretion between 5.0 and 7.9 mmol/24 h occurred in 37% of patients and ≥8.0 mmol/24 h in 48% of patients. Although the number of patients with urinary calcium excretion ≥8.0 mmol/24 h declined significantly under the balanced mixed diet, urinary calcium excretion remained ≥5 mmol/24 h in 80% of patients. Urinary phosphate excretion ≥35 mmol/24 h was diagnosed in 43% of brushite stone formers on their habitual, self-selected diet, but only in 11% of patients during the intake of the balanced mixed diet. While 17% of brushite stone patients had hyperoxaluria under the usual diet, only one patient presented hyperoxaluria under the balanced, standardized diet. The number of brushite stone patients with hypocitraturia, elevated urinary pH, and low urine volume was similar in both study phases.

### 3.3. [^13^C_2_]Oxalate Absorption Test

Intestinal hyperabsorption of oxalate, defined as [^13^C_2_]oxalate absorption ≥10%, was diagnosed in 41% of the patients (Table 1). Hyperabsorption of oxalate was similarly common in men (*n* = 10/25; 40.0%) and women (*n* = 4/9; 44.4%) (*p* = 1.000). Intestinal oxalate absorption correlated significantly positively with urinary oxalate excretion on the balanced mixed diet (R = 0.472; *p* = 0.005) but not on the habitual, free-choice diet (R = 0.075; *p* = 0.674).

### 3.4. Ammonium Chloride Loading Test

Of the 62 patients who underwent the ammonium chloride loading test, 50% were diagnosed with dRTA (Table 1), with women being affected significantly more frequently than men (women: *n* = 13/18, 72.2%; men: *n* = 18/44; 40.9%; *p* = 0.049). Out of 13 women, 2 had complete dRTA. Urinary pH was significantly higher in patients with dRTA than in patients without dRTA, both under the habitual diet and under the balanced diet (Table 4). Under both diets, urinary excretion of calcium, phosphate, and citrate was significantly lower in patients with dRTA than in patients without dRTA. No statistically significant difference was detected between stone formers with and without dRTA in the relative supersaturation of brushite and the ion-activity product of brushite, both under the habitual diet and under the balanced mixed diet. The relative supersaturation of struvite was significantly higher in patients with dRTA than in patients without dRTA on both diets. Urinary density, chloride and creatinine excretion, the ion-activity product of uric acid, and the relative supersaturation of uric acid were significantly lower in patients with dRTA, while the ion-activity product of struvite was significantly higher in patients with dRTA, but only under one of the two diets. No difference was found in the other urinary parameters.

### 3.5. Calcium Loading Test

Of the 55 brushite stone patients with a urinary calcium excretion ≥5 mmol/24 h under the usual diet, calcium loading test results were available in 53 patients (Table 5). Absorptive hypercalciuria was present in 32.1% and renal hypercalciuria in 28.3%, while idiopathic hypercalciuria was noted in 39.6% of brushite stone formers. Urinary calcium excretion was highest in patients with renal hypercalciuria, moderate in absorptive hypercalciuria, and lowest in idiopathic hypercalciuria under the balanced diet, while no significant difference was observed under the habitual diet. In addition, urinary calcium excretion was significantly lower under the balanced diet than under the habitual, free-choice diet for any type of hypercalciuria.

### 3.6. Diet Composition

The diet composition of the habitual, free-choice diet and the balanced, standardized diet is given in Table 6. The mean daily intakes of carbohydrates, fiber, potassium, calcium, phosphate, and oxalate were significantly higher, while the mean daily intakes of protein, sodium, cystine, methionine, and cholesterol were significantly lower with the balanced mixed, standardized diet. The consumption of alcohol was not permitted during the balanced, controlled and standardized diet.

## 4. Discussion

Brushite calculi are highly recurrent, particularly dense and hard, and resistant to ESWL [1,3,5,26,27]. The present study confirmed significant stone activity in brushite stone patients and associated numerous interventions. In the 12 months prior to admission, patients had passed an average of nine urinary stones. The mean duration of urinary stone disease at the time of enrollment was 12 years. All but one of the patients have had some form of surgical treatment during their lifetime. The majority of patients, i.e., 68%, had formed bilateral stones, which is twice the previously reported frequency of 34% [7]. 

All brushite stone patients presented with at least one urinary abnormality on the usual diet. The most common abnormality in the 24 h urine of brushite stone formers in the present cohort was hypercalciuria. Hypercalciuria, defined as urinary calcium excretion ≥5 mmol/24 h, was diagnosed in 85% of patients on their habitual, self-selected diet. Although urinary calcium excretion decreased significantly under the balanced diet, hypercalciuria was still present in 80% of patients. These results confirmed previous findings that hypercalciuria is the predominant abnormality in 24 h urine in brushite stone formers [7,10,11]. Various etiologic factors may be associated with hypercalciuria, including dietary factors, dRTA, primary hyperparathyroidism, enhanced intestinal absorption, and decreased renal tubular reabsorption of calcium (“renal leak”) [28,29]. In the present study, dRTA was identified in 50% of the patients, which is consistent with previous findings in 39 brushite stone formers [30]. Because urinary calcium excretion in patients with dRTA was significantly lower than in patients without dRTA on both the usual, free-choice diet and the balanced, standardized diet, it is assumed that dRTA was not responsible for the hypercalciuria in our cohort of brushite stone patients.

Absorptive hypercalciuria has been commonly encountered in brushite stone disease [30,31]. To our knowledge, this study is the largest series of brushite stone patients, distinguishing between different types of hypercalciuria. Idiopathic hypercalciuria was diagnosed in 39.6%, absorptive hypercalciuria in 32.1%, and renal hypercalciuria in 28.3% of patients. Urinary calcium excretion differed significantly between the different types of hypercalciuria under the balanced, standardized diet and was lowest in idiopathic hypercalciuria, moderate in absorptive hypercalciuria, and highest in renal hypercalciuria. Since urinary calcium excretion in all types of hypercalciuria was significantly lower under the balanced mixed diet than under the habitual, free-choice diet, it is assumed that diet exerts a substantial effect on urinary calcium excretion.

Several dietary factors have been reported to influence urinary calcium excretion, especially high intakes of protein, sodium chloride, and calcium [16,32,33]. Because the calcium content of the balanced mixed diet was significantly higher than that of the habitual diet, it is hypothesized that the significant decrease in urinary calcium excretion was due to the significantly lower intake of protein and sodium chloride with the balanced mixed, standardized diet. It is noteworthy that the mean calcium intake of brushite stone patients with the usual diet was only 837 mg/day, which was markedly lower than the dietary recommendation of 1000–1200 mg calcium per day. In contrast, sodium intake with the usual, free-choice diet was almost 75% higher than with the balanced mixed, standardized diet. The present results suggest that intestinal hyperabsorption and reduced renal tubular reabsorption (“renal leak”) of calcium have a considerable impact on urinary calcium excretion, but that dietary factors, especially protein, sodium chloride, and calcium, can also significantly modify urinary calcium excretion.

Interestingly, urinary phosphate excretion decreased significantly under the balanced mixed diet, although the phosphate content was significantly higher than that of the usual diet. Furthermore, the frequency of hyperphosphaturia declined significantly from 43% under the habitual diet to 11% under the balanced mixed diet, although phosphate intake was higher with the balanced, standardized diet than with the habitual, free-choice diet. The present results suggest that hyperphosphaturia was mainly determined by dietary factors, particularly increased bioavailability of dietary phosphate due to lower calcium intake [34]. Since urinary phosphate excretion was significantly lower in patients with dRTA than in patients without dRTA under both diets, it is assumed that dRTA did not contribute to hyperphosphaturia in our patient cohort.

A major risk factor for brushite stone formation is urine pH. Brushite crystallizes at a urine pH in the range of 6.5–6.8 and can convert to carbonate apatite at pH 6.9 and above [1,35]. In this study, mean urine pH did not change under the balanced mixed diet and remained above 6.5. From these results, it can be concluded that the urine pH of patients with brushite nephrolithiasis is not changed by diet. Urine pH was significantly higher in brushite stone formers with dRTA than in patients without dRTA, both under the habitual, self-selected diet and the balanced, standardized diet. Despite a significantly higher urine pH in patients with dRTA than in patients without dRTA, the relative supersaturation of brushite and the ion-activity product of brushite were similar in patients with dRTA and without dRTA under both diets, which can be attributed to the significantly lower urinary calcium and phosphate excretion. The findings suggest that the presence of dRTA does not necessarily indicate a higher risk of brushite stone formation. Further research on the impact of dRTA on urinary risk factors for brushite stone formation is needed.

Diminished urinary citrate excretion is another common metabolic abnormality observed in patients with brushite stones. In the present study, urinary citrate excretion differed between the habitual, self-selected diet and the balanced, standardized diet. Hypocitraturia, defined as urinary citrate excretion below 1.7 mmol/24 h, was present in 32% of patients under the habitual diet and in 39% of patients under the balanced mixed diet. The present results confirm previous findings in 45 brushite stone formers, 46.8% of whom had urinary citrate excretion of less than 320 mg/24 h [7]. Remarkably, urinary citrate excretion was significantly lower in patients with dRTA under both diets than in patients without dRTA. In the absence of dietary factors, it is hypothesized that the reduced urinary citrate excretion observed in this study is due to acid retention causing intracellular acidosis in proximal tubule cells, which promotes renal tubule citrate reabsorption [36,37].

In this study, urinary oxalate excretion declined significantly under the balanced mixed diet, despite significantly higher dietary oxalate intake. It is suggested that the higher calcium content of the balanced diet resulted in the formation of insoluble complexes with oxalate in the intestine, reducing absorption and urinary excretion of oxalate [38]. While 17% of brushite stone patients had hyperoxaluria under the habitual, free-choice diet, only one patient presented hyperoxaluria under the balanced mixed diet. Furthermore, intestinal oxalate absorption correlated significantly positively with urinary oxalate excretion under the balanced mixed diet. Although increased urinary oxalate excretion is not considered a risk factor for brushite stone formation, it could promote the formation of mixed calculi with calcium oxalate [11].

Remarkably, the majority of patients in our cohort, i.e., 62%, had mixtures of brushite with other constituents in the stone analysis, with calcium oxalate being the most common secondary stone constituent among patients in the current series. Of all brushite-containing calculi, 35% were mixed with calcium oxalate, 14% with carbonate apatite, and 12% with calcium oxalate and carbonate apatite. These results are consistent with previous findings that brushite is often mixed with another stone component [4,7]. Although brushite stone disease differs greatly from idiopathic calcium oxalate stone disease, many brushite stone patients have been found to have initially formed calcium oxalate calculi [39,40,41]. It has been hypothesized that brushite and calcium oxalate stone patients share a common pathogenesis [39,40]. Studies suggested that with recurrent stone episodes, the conversion of calcium oxalate to brushite or carbonate apatite stone disease may occur over time [39,40]. It is presumed to be the result of external injury to the nephron, either by natural obstruction or by iatrogenic effects such as ESWL or alkalization therapy [40,42]. Further research on the relationship between brushite stones and ESWL is necessary.

Metabolic evaluation of brushite stone patients revealed a high risk of brushite and calcium oxalate stone formation on the habitual, self-selected diet. The ion-activity product of brushite declined significantly by 26% on the balanced mixed diet, mainly due to the significant decline in urinary calcium and phosphate excretion. In addition, the ion-activity product of calcium oxalate decreased significantly and to the same extent, which can be attributed to a significant decline in urinary calcium and oxalate excretion. The decline in the relative supersaturation of brushite and calcium oxalate confirmed these findings. Moreover, the relative supersaturation of apatite decreased significantly by 59%. Because a urinary volume of less than 2.0 L per 24 h was observed in 26% of patients under the habitual, free-choice diet and in 23% of patients under the balanced mixed diet, major attention should be paid to adequate urinary dilution. Nutritional counselling should ascertain that patients achieve a urine volume of at least 2.0 to 2.5 L per 24 h and compensate for exceptional fluid losses. In the context of nutritional counselling, self-monitoring of urine volume is an essential general recommendation for the efficient treatment of brushite nephrolithiasis. In this study, dietary intervention was shown to be effective and should be an integral part of the management of brushite stone disease. 

## 5. Conclusions

Brushite stone disease is a malignant form of urolithiasis that not only warrants an aggressive treatment approach but also requires comprehensive metabolic evaluation for effective recurrence prevention. All brushite stone patients in this cohort had at least one metabolic abnormality on the usual diet, with hypercalciuria and urine pH above 6.5 being the most common. Urinary calcium excretion was lowest in idiopathic hypercalciuria, moderate in absorptive hypercalciuria, and highest in renal hypercalciuria, differing significantly between the different types of hypercalciuria under the balanced, standardized diet. Moreover, urinary calcium excretion in all types of hypercalciuria was significantly lower under the balanced mixed diet than under the habitual diet. The current study is, to our knowledge, the first study to investigate the impact of a dietary intervention under controlled, standardized conditions on the metabolic risk profile of brushite stone formers. The findings of this study suggest that brushite stone patients benefit from a balanced mixed diet. The relative supersaturation of brushite, calcium oxalate, and apatite declined significantly under the balanced mixed diet compared to the usual diet, mainly due to the significant reduction in urinary calcium, phosphate, and oxalate excretion. Further research on the role of primary hyperparathyroidism and dRTA in brushite stone formation is necessary.

## Figures and Tables

**Table 1:** Characteristics of brushite stone patients.

	Mean ± SD *n* (%)
Number of patients	65
Gender (men/women)	46/19
Age (years)	41.9 ± 13.0
BMI (kg/m^2^) ^a^	26.3 ± 4.3
BMI < 18.5 kg/m^2 a^	2/64 (3.1)
BMI 18.5–24.9 kg/m^2 a^	27/64 (42.2)
BMI 25.0–29.9 kg/m^2 a^	24/64 (37.5)
BMI > 30.0 kg/m^2 a^	11/64 (17.2)
Type 2 diabetes ^a^	2/64 (3.1)
Hypertension ^a^	14/64 (21.9%)
[^13^C_2_]Oxalate absorption (%) ^b^	9.8 ± 6.5
[^13^C_2_]Oxalate absorption < 10% ^b^	20/34 (58.8)
[^13^C_2_]Oxalate absorption ≥ 10% ^b^	14/34 (41.2)
Hypercalciuria	55/65 (84.6)
Idiopathic hypercalciuria ^c^	21/53 (39.6)
Absorptive hypercalciuria ^c^	17/53 (32.1)
Renal hypercalciuria ^c^	15/53 (28.3)
Distal renal tubular acidosis (dRTA) ^d^	31/62 (50.0)
Complete dRTA ^d^	2/62 (3.2)
Incomplete dRTA ^d^	29/62 (46.8)
Glomerular filtration rate (mL/min/1.73 m^2^) ^a^	91.5 ± 19.5
Family history of urolithiasis ^e^	29/58 (50.0)
Age at first stone (years) ^a^	29.9 ± 11.9
Duration of stone disease (years) ^a^	11.8 ± 9.6
Stone passages in the last 12 months ^f^	9 ± 15
Stone passages total ^g^	38 ± 81
Type of stone removal ^a^	
Spontaneous passage (patients) ^a^	48/64 (75.0)
Ureteroscopy (patients) ^a^	55/64 (85.9)
Extracorporeal shock wave lithotripsy (patients) ^a^	53/64 (82.8)
Percutaneous nephrolithotomy (patients) ^a^	28/64 (45.3)
Open surgery (patients) ^a^	15/64 (23.4)
Laterality ^h^	
Bilateral	42/62 (67.7)
Right	9/62 (14.5)
Left	11/62 (17.7)
Anatomical abnormalities ^a^	12/64 (18.8)
Kidney cysts ^a^	4/64 (6.3)
Stenosis ^a^	4/64 (6.3)
Ureteral duplication ^a^	1/64 (1.6)
Medullary sponge kidney ^a^	3/64 (4.7)

Abbreviations: BMI, body mass index; dRTA, distal renal tubular acidosis; SD, standard deviation; ^a^ *n* = 64 (19 women, 45 men); ^b^ *n* = 34 (9 women, 25 men); ^c^ *n* = 53/55 (14 women, 39 men); ^d^ *n* = 62 (18 women, 44 men); ^e^ *n* = 58 (19 women, 39 men); ^f^ *n* = 61 (18 women, 43 men); ^g^ *n* = 55 (16 women, 39 men); ^h^ *n* = 62 (19 women, 43 men).

**Table 2:** Urinary parameters under the habitual diet and the balanced diet.

	Habitual Diet *n* = 65 Mean ± SD	Balanced Diet *n* = 65 Mean ± SD	*p* Value
Volume (L/24 h)	2.494 ± 0.795	2.400 ± 0.526	0.388
Density (g/cm^3^)	1.009 ± 0.004	1.006 ± 0.003	<0.001
Urinary pH	6.57 ± 0.30	6.54 ± 0.31	0.410
Sodium (mmol/24 h)	179 ± 65	102 ± 33	<0.001
Potassium (mmol/24 h)	65 ± 21	55 ± 16	<0.001
Calcium (mmol/24 h)	8.20 ± 3.09	7.12 ± 2.73	<0.001
Magnesium (mmol/24 h)	5.17 ± 1.87	4.90 ± 1.42	0.188
Ammonium (mmol/24 h) ^a^	27.8 ± 10.6	28.4 ± 9.8	0.753
Chloride (mmol/24 h)	183 ± 73	109 ± 32	<0.001
Phosphate (mmol/24 h)	32.7 ± 9.0	27.2 ± 6.5	<0.001
Sulfate (mmol/24 h)	21.8 ± 6.6	17.7 ± 3.7	<0.001
Creatinine (mmol/24 h)	15.05 ± 4.14	14.55 ± 3.84	0.060
Uric acid (mmol/24 h)	3.88 ± 1.10	3.32 ± 0.76	<0.001
Oxalate (mmol/24 h)	0.383 ± 0.120	0.303 ± 0.077	<0.001
Citrate (mmol/24 h)	2.257 ± 1.430	2.092 ± 1.212	0.612
AP Uric acid (×10^9^)	0.33 ± 0.39	0.30 ± 0.35	0.933
AP Calcium oxalate index	1.29 ± 0.58	0.96 ± 0.52	<0.001
AP Struvite index ^a^	11.73 ± 13.88	8.53 ± 7.29	0.083
AP Brushite index	8.51 ± 4.94	6.33 ± 3.39	0.001
RS Uric acid	0.50 ± 0.58	0.47 ± 0.52	0.965
RS Calcium oxalate	5.81 ± 2.58	5.01 ± 2.61	0.006
RS Struvite ^a^	0.17 ± 0.18	0.14 ± 0.13	0.516
RS Apatite (×10^−28^)	1.51 ± 3.37	0.62 ± 1.57	0.005
RS Brushite	2.01 ± 0.82	1.68 ± 0.70	0.002

Abbreviations: AP, ion-activity product; RS, relative supersaturation; SD, standard deviation; ^a^ *n* = 63 (17 women, 46 men).

**Table 3:** Urinary abnormalities under the habitual diet and the balanced diet.

	Reference Range	Habitual Diet *n* = 65 *n* (%)	Balanced Diet *n* = 65 *n* (%)	*p* Value
Volume (L/24 h)	<2.000	17 (26.2)	15 (23.1)	0.832
	≥2.000	48 (73.8)	50 (76.9)	0.832
Urinary pH	<6.50	25 (38.5)	23 (35.4)	0.824
	6.50–6.79	27 (41.5)	31 (47.7)	0.523
	≥6.80	13 (20.0)	11 (16.9)	0.774
Phosphate (mmol/24 h)	<35.0	37 (56.9)	58 (89.2)	<0.001
	≥35.0	28 (43.1)	7 (10.8)	<0.001
Calcium (mmol/24 h)	<5.0	10 (15.4)	13 (20.0)	0.508
	5.0–7.9	24 (36.9)	32 (49.2)	0.185
	≥8.0	31 (47.7)	20 (30.8)	0.019
Oxalate (mmol/24 h)	<0.500	54 (83.1)	64 (98.5)	0.002
	≥0.500	11 (16.9)	1 (1.5)	0.002
Citrate (mmol/24 h)	<1.700	21 (32.3)	25 (38.5)	0.344
	≥1.700	44 (67.7)	40 (61.5)	0.344

**Table 4:** Urinary parameters in brushite stone patients with or without dRTA under the habitual diet and the balanced diet.

		Habitual Diet			Balanced Diet	
	dRTA *n* = 31 Mean ± SD	Non-dRTA *n* = 31 Mean ± SD	*p* Value	dRTA *n* = 31 Mean ± SD	Non-dRTA *n* = 31 Mean ± SD	*p* Value
Volume (L/24 h)	2.522 ± 0.716	2.545 ± 0.834	0.930	2.418 ± 0.519	2.407 ± 0.553	0.637
Density (g/cm^3^)	1.008 ± 0.003	1.010 ± 0.004	0.052	1.005 ± 0.003	1.006 ± 0.003	0.042
Urinary pH	6.70 ± 0.23	6.49 ± 0.31	0.011	6.65 ± 0.24	6.44 ± 0.34	0.024
Calcium (mmol/24 h)	7.06 ± 2.73	9.36 ± 3.00	0.002	6.15 ± 2.00	8.03 ± 2.48	0.001
Chloride (mmol/24 h)	175 ± 70	197 ± 73	0.173	104 ± 29	120 ± 30	0.034
Phosphate (mmol/24 h)	30.3 ± 8.5	35.1 ± 8.9	0.038	25.5 ± 4.6	29.1 ± 7.8	0.012
Creatinine (mmol/24 h)	13.96 ± 4.03	16.40 ± 3.71	0.011	13.74 ± 3.38	15.65 ± 3.83	0.059
Citrate (mmol/24 h)	1.796 ± 1.177	2.798 ± 1.478	0.006	1.488 ± 1.069	2.653 ± 0.968	<0.001
AP Uric acid (×10^9^)	0.19 ± 0.10	0.41 ± 0.48	0.042	0.21 ± 0.18	0.38 ± 0.46	0.069
AP Struvite index ^a^	16.35 ± 18.31	8.24 ± 6.68	0.042	10.70 ± 8.59	6.95 ± 5.60	0.073
AP Brushite index	8.55 ± 4.60	7.85 ± 4.32	0.529	7.19 ± 4.11	5.42 ± 2.10	0.100
RS Uric acid	0.30 ± 0.16	0.62 ± 0.71	0.042	0.34 ± 0.28	0.60 ± 0.67	0.078
RS Struvite ^a^	0.23 ± 0.23	0.12 ± 0.10	0.024	0.19 ± 0.15	0.11 ± 0.09	0.016
RS Brushite	1.85 ± 0.81	2.15 ± 0.85	0.157	1.68 ± 0.72	1.68 ± 0.59	0.695

Abbreviations: AP, ion-activity product; RS, relative supersaturation; SD, standard deviation; ^a^ *n* = 60 (29 dRTA patients, 31 non-dRTA patients).

**Table 5:** Urinary calcium excretion according to the different types of hypercalciuria under the habitual diet and the balanced diet.

	Idiopathic Hypercalciuria *n* (%)	Absorptive Hypercalciuria *n* (%)	Renal Hypercalciuria *n* (%)	*p* Value
Number of patients	21/53 (39.6)	17/53 (32.1)	15/53 (28.3)	
Gender (men/women)	17/4 (81.0/19.0)	13/4 (76.5/23.5)	9/6 (60.0/40.0)	0.353
	**Calcium** (**mmol/24 h**) **Mean ± SD**	**Calcium** (**mmol/24 h**) **Mean ± SD**	**Calcium** (**mmol/24 h**) **Mean ± SD**	***p* Value**
Habitual diet	8.30 ± 2.24	8.74 ± 2.58	10.0 ± 2.40	0.113
Balanced diet	6.79 ± 1.86	7.11 ± 1.48	8.85 ± 2.68	0.011
*p* Value	0.001	0.003	0.048	

**Table 6:** Nutrient intake under the habitual diet and the balanced diet.

	Habitual Diet *n* = 43 Mean ± SD	Balanced Diet *n* = 43 Mean	*p* Value
Energy (kcal/day)	2256 ± 485	2355	0.347
Carbohydrates (g/day)	264 ± 71	327	<0.001
Fat (g/day)	84 ± 19	81	0.237
Protein (g/day)	84 ± 19	71	<0.001
Methionine (mg/day)	1644 ± 426	1415	0.002
Cystine (mg/day)	1052 ± 267	835	<0.001
Purines (mg/day)	420 ± 143	449	0.176
Cholesterol (mg/day)	330 ± 81	195	<0.001
Fiber (g/day)	20.6 ± 7.2	31.0	<0.001
Sodium (mg/day) ^a^	4033 ± 1213	2300	<0.001
Potassium (mg/day)	3010 ± 705	3390	0.001
Calcium (mg/day)	837 ± 360	977	0.004
Magnesium (mg/day)	360 ± 113	341	0.560
Phosphate (mg/day)	1225 ± 300	1432	<0.001
Oxalate (mg/day)	111 ± 43	121	0.049
Alcohol (g/day)	10.0 ± 15.4	0	<0.001
Water (mL/day)	3462 ± 969	3437	0.507

^a^ Estimated from urinary sodium excretion.

## Data Availability

The data presented in this study are available upon reasonable personal request.
